# Quantitative assessment of mesenchymal stem cells contained in concentrated autologous bone marrow aspirate transplantation for the treatment of osteonecrosis of the femoral head: predictive factors and differences by etiology

**DOI:** 10.1186/s13104-018-3949-6

**Published:** 2018-11-29

**Authors:** Hiroshi Kumagai, Tomokazu Yoshioka, Hisashi Sugaya, Yohei Tomaru, Yukiyo Shimizu, Masashi Yamazaki, Hajime Mishima

**Affiliations:** 1 Department of Orthopaedic Surgery, Faculty of Medicine, University of Tsukuba, Tsukuba, Ibaraki Japan; 2 Department of Rehabilitation Medicine, Faculty of Medicine, University of Tsukuba, Tsukuba, Ibaraki Japan

**Keywords:** Concentrated autologous bone marrow aspirate transplantation, Osteonecrosis of the femoral head, Joint-preserving surgery, Mesenchymal stem cells

## Abstract

**Objective:**

We previously established concentrated autologous bone marrow aspirate transplantation as a one-step, lowly invasive, joint-preserving surgical technique for treating osteonecrosis of the femoral head. The objectives of this study were to identify factors that may predict the mesenchymal stem cell (MSC) count in bone marrow aspirate, concentrated using our method, and to clarify etiology related differences in the number of MSCs in concentrated bone marrow aspirate.

**Results:**

The MSC counts per 10^6^ nucleated cells before concentration in the steroid, alcohol, and trauma groups were 2.31 ± 2.96, 2.58 ± 2.30, and 1.95 ± 1.85, respectively. The MSC counts per 10^6^ nucleated cells after concentration were 3.23 ± 3.41, 3.30 ± 2.83, and 2.56 ± 1.98 cells, respectively. The MSC concentration rates in the steroid, alcohol, and trauma groups were 7.15 ± 5.62, 5.08 ± 1.96, and 8.23 ± 4.82 times, respectively. None of the differences were significant. Multiple regression analysis revealed that MSC count was related to the total bone marrow aspirated, peripheral blood platelet count, and nucleated cell count in the initial aspiration.

## Introduction

We developed a simple and efficient method for collecting mesenchymal stem cells (MSCs) by centrifuging bone marrow aspirate to concentrate and extract a buff-coat layer containing MSCs, which may be used to treat osteonecrosis of the femoral head (ONFH) [1, 2]. The main characteristics of this procedure are that it takes approximately 2 h from bone marrow aspiration to transplantation into the necrosis site and the fact that it is a one-step joint-preserving surgery.

Muschler et al. [3] reported that progenitor cells constitute approximately 1 per 30,000 nucleated cells in an iliac bone marrow aspirate. Hernigou et al. [4, 5] reported positive outcomes in the treatment of ONFH and nonunion by concentrating close to 600 progenitor cells/mL of iliac bone marrow aspirate to approximately 2500 cells/mL using a cell separator. They also reported a formula for predicting the number of nucleated cells in bone marrow as follows: *N* (10^8^/kg) = (*V* × NP) − (*V* − 100) × NS/*P*, where *V* is the total volume of aspirate, NP is the nuclear cell count per milliliter of bone marrow aspirate, NS is the nuclear cell count per ml of peripheral blood, and *P* is the patient’s weight [4].

Our method for concentrating bone marrow aspirate does not use a cell separator [1]. Previously, we reported positive outcomes gained by using this method in the treatment of ONFH [2, 6]. It has also been applied in the treatment of nonunion [7].

In the present study, we cultured a portion of the bone marrow aspirate for transplantation to estimate MSC count on the basis of fibroblastic colony- forming unit (CFU-F). However, as we did not perform cell culture or predictive screenings, we could not calculate the MSC count in bone marrow aspirate during the procedure.

Furthermore, reviewed literatures did not indicate whether differences in MSC counts were based on etiology. Approximately half (51%) of ONFH cases in Japan are steroid related [8]. Therefore, the effects of steroid therapy on the quality and quantity of bone marrow aspirates must be investigated. Predicting the number of MSCs prior to bone marrow transplantation may be useful for clarifying the efficacy and limitations of this procedure.

The objectives of this study were to identify predictive factors of and clarify etiology-related differences in MSC count in concentrated bone marrow aspirate.

## Main text

### Materials and methods

#### Patients

The study subjects were 93 patients (60 men and 33 women) who underwent joint-preserving surgery for ONFH at our hospital between November 2012 and May 2017. The etiology was steroid related in 58 patients, alcohol related in 18, and trauma related in 17. In this series, of the 93 patients, 58 (62.4%) had steroid-related ONFH and 22 (38.0%) had steroid-related ONFH with systemic lupus erythematosus (SLE). Of the 32 men and 26 women, 6 (18.8%) and 16 (61.6%) had SLE, respectively. The mean age was 41.0 ± 11.5 years (Table 1).

**Table 1:** Patient demographics

Number of enrolled patients	93
Age range	41.0 ± 11.5
Male:female	60:33
Average BMI	23.3 ± 3.4
Etiology	
Steroid related	58 (M: 32 F: 26)^a^
Alcohol related	18 (M: 17 F: 1)
Trauma related	17 (M: 11 F: 6)

^a^Steroid related patient with SLE 24 (M: 7 F: 17), without SLE 34 (M: 25 F: 9)

#### Bone marrow aspiration, concentration, and transplantation

Bone marrow aspiration, concentration, and transplantation were performed using the method previously developed by Yoshioka et al. and Tomaru et al. [5, 6]. Firstly, bone marrow was aspirated from both anterior iliac crests using a bone marrow harvesting needle. The bone marrow aspirates were processed by a two-step centrifugation method at room temperature. The bone marrow concentrates containing buffy coat were extracted. This technique reduced the typical 300 mL of bone marrow aspirate to bone marrow concentrates of approximately 30–40 mL.

Before transplantation, multidirectional holes are made to perforate the interface between the areas of ONFH by drilling with a Kirschner wire. Under bi-plane fluoroscopic control, the transplantation was performed. After the operation, weight bearing was limited for 6 weeks, while non-weight bearing exercise was allowed.

#### Bone marrow aspirate evaluation

During the operation, 2 mL of bone marrow aspirate containing anticoagulant citrate dextrose solution was collected from the bag, and another 2 mL was collected after being concentrated for culturing. Bone marrow aspirate samples were mixed with 8 ml of phosphate-buffered saline (PBS) and centrifuged, and 9 mL of the supernatant was removed. Next, 4 mL of culture medium was added and 500-µL portions of a 5-mL total were seeded into 6-well dishes.

Each dish was seeded with 100 µL of bone marrow aspirate, and each plate was seeded with 600 µL of bone marrow aspirate, which was cultured at 37 °C in 5% carbon dioxide. The culture medium comprised of Dulbecco’s modified Eagle’s medium (Sigma, St. Louis, MO, USA), 10% fetal bovine serum (Gibco, Grand Island, NY, USA), and 1% antibiotic–antimycotic solution (Gibco). Each plate was filled with 2 mL of the medium, and the first medium replacement was performed 24 h later. Thereafter, the medium was replaced every 2–3 days, and crystal-violet staining was performed when the colonies could not be distinguished because of overlapping, or 14 days after seeding. Washing was conducted twice with PBS, and 2 ml of crystal violet was added to each dish. The mixture was left to stand for 5 min. The specimens were then rinsed with water, dried, and examined with a microscope. Colonies with diameters of ≥ 2 mm were counted to obtain the number of CFU-F. The mean of two counts was used as the measurement.

For this study, MSC count was defined as the CFU-F count. Total bone marrow aspirated, nucleated cell count (peripheral blood and initial aspiration in bag after concentration), red blood cell (RBC) count (in bag after concentration), platelet count (in bag after concentration), and MSC count (in bag after concentration) were measured. Rates of concentration from the bone marrow aspirate in the bag to the concentrated bone marrow aspirate were calculated (nucleated cells, RBC, platelets and MSCs). We investigated whether the MSC count after concentration had sex-related differences. Among the patients with steroid-related ONFH, we compared the MSC count after concentration regardless of SLE.

Etiology-based differences in the specimens were statistically examined using the Student *t* test. By using the concentrated MSC count as the dependent variable, multiple regression analysis was performed to determine factors (independent variables) that may predict the concentrated MSC count. The independent variables were age, body mass index, nucleated cell count (peripheral blood and initial aspiration in bag after concentration), RBC count (peripheral blood and initial aspiration after concentration), platelet count (peripheral blood and initial aspiration after concentration), peripheral blood fraction, nucleated cell concentration rate, RBC concentration rate, platelet concentration rate, and total bone marrow aspirated.

### Results

The total bone marrow aspirated was 252.5 ± 83.1 mL in the steroid group, 244.7 ± 81.9 mL in the alcohol group, and 280.3 ± 51.9 mL in the trauma group. The nucleated cell counts in the steroid, alcohol, and trauma groups in peripheral blood were 6.9 ± 2.9 × 10^3^/μL, 5.9 ± 1.4 × 10^3^/μL, and 6.0 ± 2.1 × 10^3^/μL, respectively. The nucleated cell counts in the initial aspiration were 51.4 ± 28.5 × 10^3^/μL, 53.2 ± 25.6 × 10^3^/μL, and 46.4 ± 22.9 × 10^3^/μL, respectively. The nucleated cell counts in the bag were 10.5 ± 5.1 × 10^3^/μL, 9.4 ± 3.6 × 10^3^/μL, and 10.4 ± 3.8 × 10^3^/μL, respectively. The nucleated cell counts after concentration were 40.3 ± 23.6 × 10^3^/μL, 39.2 ± 16.0 × 10^3^/μL, and 48.3 ± 20.4 × 10^3^/μL, respectively.

The nucleated cell concentration rates in the steroid, alcohol, and trauma groups were 4.1 ± 1.9, 4.5 ± 1.7, and 4.8 ± 1.9 times, respectively. The RBC concentration rates were 1.2 ± 0.5, 1.0 ± 0.3, and 1.0 ± 0.3 times, respectively. The platelet concentration rates were 6.2 ± 2.2, 6.1 ± 1.9, and 7.5 ± 2.5 times, respectively.

The MSC counts per 10^6^ nucleated cells before concentration in the steroid, alcohol, and trauma groups were 2.31 ± 2.96, 2.58 ± 2.30, and 1.95 ± 1.85 cells, respectively. The MSC counts per 10^6^ nucleated cells after concentration were 3.23 ± 3.41, 3.30 ± 2.83, and 2.56 ± 1.98 cells, respectively.

Per milliliter of blood marrow aspirate, the counts before concentration in the steroid, alcohol, and trauma groups were 23.09 ± 28.22, 23.94 ± 19.31, and 19.22 ± 21.74 cells, respectively. The counts after concentration were 137.41 ± 160.06, 129.04 ± 113.95, and 103.99 ± 54.71 cells, respectively. The MSC concentration rates in the steroid, alcohol, and trauma groups were 7.15 ± 5.62, 5.08 ± 1.96, and 8.23 ± 4.82 times, respectively. None of these differences were significant (Table 2).

**Table 2:** Cell count according to etiology

	Corticosteroid treatment	Alcohol abuse	Trauma
Total bone marrow aspirated (mL)	252.5 ± 83.1	244.7 ± 81.9	280.3 ± 52.0
Nucleated cell count: peripheral blood (× 1000/μL)	6.9 ± 2.9	5.9 ± 1.4	6.0 ± 2.1
Nucleated cell count: initial aspiration (× 1000/μL)	51.4 ± 28.5	53.2 ± 25.6	46.4 ± 22.9
Nucleated cell count: before concentration (× 1000/μL)	10.5 ± 5.1	9.4 ± 3.6	10.4 ± 3.8
Nucleated cell count: after concentration (× 1000/μL)	40.3 ± 23.6	39.2 ± 16.0	48.3 ± 20.4
Nucleated cell count concentration rate (times)	4.1 ± 1.9	4.5 ± 1.7	4.8 ± 1.9
Red blood cell concentration rate (times)	1.2 ± 0.5	1.0 ± 0.3	1.0 ± 0.3
Platelet concentration rate (times)	6.2 ± 2.2	6.1 ± 1.9	7.5 ± 2.5
MSC count: before concentration (per 10^6^ nucleated cells)	2.3 ± 3.0	2.6 ± 2.3	2.0 ± 1.9
MSC count: after concentration (per 10^6^ nucleated cells)	3.2 ± 3.4	3.3 ± 2.8	2.6 ± 2.0
MSC count: before concentration (per 1 mL bone marrow aspirate)	23.1 ± 28.2	23.9 ± 19.3	19.2 ± 21.7
MSC count: after concentration (per 1 mL bone marrow aspirate)	137.4 ± 160.1	129.0 ± 114.0	104.0 ± 54.7
MSC concentration rate (times)	7.2 ± 5.6	5.1 ± 2.0	8.2 ± 4.8

None of these differences were significant

The mean MSC counts after concentration in the men (151.3 ± 146.6 cells) was higher than that in the women (89.4 ± 108.8 cells) with a significant difference (*p *< 0.025). The mean MSC count after concentration in the patients with steroid-related ONFH patients with SLE (95.3 ± 97.3 cells) was lower than that in the patients with steroid-related ONFH without SLE (164.7 ± 95.3 cells; *p *= 0.114). Multiple regression analysis indicated that the MSC count was related to the total bone marrow aspirated, peripheral blood platelet count, and nucleated cell count in the initial aspiration (Table 3).

**Table 3:** Multiple regression analysis indicated that the MSC count

	Unstandardized coefficient	Standard error	Standardized coefficient β	t value	p value	Multicollinearity statistics tolerance
(Constant)	− 164.486	42.669		− 3.855	0	
Total bone marrow aspirated	0.475	0.104	0.426	4.563	0	0.941
Platelet count: peripheral blood	0.4	0.138	0.267	2.897	0.005	0.968
Nucleated cell count: initial bone marrow aspiration	0.617	0.303	0.191	2.033	0.005	0.93

### Discussion

Transplantation of concentrated bone marrow aspirate for ONFH was first reported by Hernigou et al. [9]. Since 2003, our department has used a simplified method for concentrating bone marrow aspirate [1] and a one-step surgical procedure involving transplanting into the area of necrosis (concentrated autologous bone marrow aspirate transplants).

In the present study, etiology-based differences in MSC count were not observed. Kato et al. [10] examined the osteogenic differentiation capacity of adipose tissue-derived MSCs in patients with steroid-induced ONFH and reported that steroid therapy may increase Dkk-1 expression level, which may reduce osteogenic differentiation capacity. Recent studies indicate that microRNA expression may affect osteogenic differentiation capacity [11, 12]. Chen et al. [13] reported that miR-708 may markedly suppress osteogenic and adipogenic differentiations of MSCs. On the other hand, the mean MSC count after concentration in the men was higher than that in the women. Jones et al. [14] indicated no significant difference in MSC count per milliliter of bone marrow aspirate between age-matched male and female subjects. In this study, a large difference was observed in the proportion of patients with steroid-related ONFH with SLE depending on their sex. Although no significant difference was found, the mean MSC count after concentration in the patients with steroid-related ONFH with SLE was lower than that in those with steroid-related ONFH without SLE. The relationship between SLE and MSC count is unknown. Caplan reported that autologous MSCs appeared to exhibit anti-inflammatory and immunomodulatory effects after transplantation for SLE [15]. It will also be important in the future to elucidate the links between SLE and MSC count.

Furthermore, the predictive factors assessed in the present study were total bone marrow aspirated, peripheral blood platelet count, and nucleated cell count in the initial aspiration. Hernigou et al. [4] reported that total bone marrow aspirated, nucleated cell count per milliliter of bone marrow aspirate, nucleated cell count per milliliter of peripheral blood, and body weight were factors that may predict the number of nucleated cells in bone marrow. These findings differ slightly from ours, which may be due to differences in the collection method for MSCs, as a cell separator was used by Hernigou et al.

If it were possible to predict the MSC count using preoperative test findings, it may be helpful in deciding on the type of surgical intervention needed. However, our study indicates that such a prediction would be difficult without resorting to bone marrow aspiration.

Some patients in the present study exhibited relatively low MSC counts. Examining individual cases and continuing to search for predictive factors may be important in achieving stable cellular therapies.

In conclusion, the post-concentration MSC count was related to the total bone marrow aspirated, peripheral blood platelet count, and nucleated cell count in the initial aspiration. Significant etiology-based differences were not observed in the MSC counts after concentration or MSC concentration rates, indicating that this collection method may be useful in the treatment of ONFH.

## Limitation

A limitation of the present study was that while the blood count and CFU-F were measured, differentiation capacity, proliferation capacity, or other cellular characteristics were not evaluated. We did not perform flow cytometry analysis for all the patients, only in some patients, and cited previous literatures to describe our method [16].

Moreover, as only patients with ONFH were included in the study, whether similar results may be observed in heavy steroid users who do not exhibit ONFH remains unclear. Therefore, it may be useful to evaluate mid- and long-term clinical outcomes based on etiology.

## Abbreviations

ONFH: osteonecrosis of the femoral head
MSC: mesenchymal stem cell
CFU-F: fibroblastic colony-forming unit
SLE: systemic lupus erythematosus
PBS: phosphate-buffered saline
RBC: red blood cell

## References

[1] Sakai S, Mishima H, Ishii T, Akaogi H, Yoshioka T, Uemura T (2008). Concentration of bone marrow aspirate for osteogenic repair using simple centrifugal methods. Acta Orthop.

[2] Yoshioka T, Mishima H, Akaogi H, Sakai S, Li M, Ochiai N (2011). Concentrated autologous bone marrow aspirate transplantation treatment for corticosteroid-induced osteonecrosis of the femoral head in systemic lupus erythematosus. Int Orthop.

[3] Muschler GF, Boehm C, Easley K (1997). Aspiration to obtain osteoblast progenitor cells from human bone marrow: the influence of aspiration volume. J Bone Joint Surg Am.

[4] Hernigou P, Poignard A, Manicom O, Mathieu G, Rouard H (2005). The use of percutaneous autologous bone marrow transplantation in nonunion and avascular necrosis of bone. J Bone Joint Surg Br.

[5] Hernigou P, Poignard A, Beaujean F, Rouard H (2005). Percutaneous autologous bone-marrow grafting for nonunions. Influence of the number and concentration of progenitor cells. J Bone Joint Surg Am.

[6] Tomaru Y, Yoshioka T, Sugaya H, Aoto K, Wada H, Akaogi H (2017). Hip preserving surgery with concentrated autologous bone marrow aspirate transplantation for the treatment of asymptomatic osteonecrosis of the femoral head: retrospective review of clinical and radiological outcomes at 6 years postoperatively. BMC Musculoskelet Disord.

[7] Sugaya H, Mishima H, Aoto K, Li M, Shimizu Y, Yoshioka T (2014). Percutaneous autologous concentrated bone marrow grafting in the treatment for nonunion. Eur J Orthop Surg Traumatol.

[8] Kubo T, Ueshima K, Saito M, Ishida M, Arai Y, Fujiwara H (2016). Clinical and basic research on steroid-induced osteonecrosis of the femoral head in Japan. J Orthop Sci.

[9] Hernigou P (2002). Treatment of osteonecrosis with autologous bone marrow grafting. Clin Orthop Relat Res.

[10] Kato T, Khanh VC, Sato K, Kimura K, Yamashita T, Sugaya H (2018). Elevated expression of Dkk-1 by glucocorticoid treatment impairs bone regenerative capacity of adipose tissue-derived mesenchymal stem cells. Stem Cells Dev.

[11] Bian Y, Qian W, Li H, Zhao RC, Shan WX, Weng X (2015). Pathogenesis of glucocorticoid-induced avascular necrosis: a microarray analysis of gene expression in vitro Int J Mol Med.

[12] Li T, Li H, Li T, Fan J, Zhao RC, Weng X (2014). MicroRNA expression profile of dexamethasone-induced human bone marrow-derived mesenchymal stem cells during osteogenic differentiation. J Cell Biochem.

[13] Hao C, Yang S, Xu W, Shen JK, Ye S, Liu X (2016). MiR-708 promotes steroid-induced osteonecrosis of femoral head, suppresses osteogenic differentiation by targeting SMAD3. Sci Rep.

[14] Jones E, Schafer R (2015). Where is the common ground between bone marrow mesenchymal stem/stromal cells from different donors and species?. Stem Cell Res Ther.

[15] Caplan AI (2009). Why are MSCs therapeutic? New data: new insight. J Pathol.

[16] Sugaya H, Yoshioka T, Kato T, Taniguchi Y, Kumagai H, Hyodo K (2018). Comparative analysis of cellular and growth factor composition in bone marrow aspirate concentrate and platelet-rich plasma. Bone Marrow Res.

